# Seasonal influence over serum and urine metabolic markers in submariners during prolonged patrols

**DOI:** 10.14814/phy2.12494

**Published:** 2015-08-11

**Authors:** Xavier Holy, Laurent Bégot, Sylvie Renault, Xavier Butigieg, Catherine André, Dominique Bonneau, Gustave Savourey, Jean-Marc Collombet

**Affiliations:** 1 Département des Services, IRBABrétigny-sur-Orge Cedex, France; 2 Département Soutien Médico-Chirurgical des Forces, IRBABrétigny-sur-Orge Cedex, France; 3 Département des Facteurs Humains, IRBA Antenne La TroncheLa Tronche Cedex, France

**Keywords:** Hypercapnia, hypovitaminosis D, metabolic markers, submariners

## Abstract

Within the framework of earlier publications, we have consistently dedicated our investigations to eliciting the effects of both seasonal vitamin D deficiency and submarine-induced hypercapnia on serum parameters for acid–base balance and bone metabolism in submariners over a 2-month winter (WP) or summer (SP) patrols. The latest findings reported herein, contribute further evidence with regard to overall physiological regulations in the same submariner populations that underwent past scrutiny. Hence, urine and blood samples were collected in WP and SP submariners at control prepatrol time as well as on submarine patrol days 20, 41, and 58. Several urine and serum metabolic markers were quantified, namely, deoxypyridinoline (DPD), lactate, albumin, creatinine, nonesterified fatty acids (NEFA), and ionized sodium (Na^+^) or potassium (K^+^), with a view to assessing bone, muscle, liver, or kidney metabolisms. We evidenced bone metabolism alteration (urine DPD, calcium, and phosphorus) previously recorded in submarine crewmembers under prolonged patrols. We also highlighted transitory modifications in liver metabolism (serum albumin) occurring within the first 20 days of submersion. We further evidenced changes in submariners’ renal physiology (serum creatinine) throughout the entire patrol time span. Measurements of ionic homeostasis (serum Na^+^ and K^+^) displayed potential seasonal impact over active ionic pumps in submariners. Finally, there is some evidence that submersion provides beneficial conditions prone to fend off seasonal lactic acidosis (serum lactate) detected in WP submariners.

## Introduction

Submariners subjected to prolonged patrols usually encounter extreme environmental conditions prone to alter basic physiological and metabolic pathways in humans. Such extreme challenging conditions involve long-term confinement, limited physical activity, modified dietary lifestyle, elevated CO_2_ atmosphere, and lack of sunlight exposure (Davies and Morris 1979). Among these distinctive features, hypercapnia ranks first as it is induced by the continuous elevated CO_2_ level (20–30 times higher than the 0.03% CO_2_ concentration in the atmospheric air).

Exposure to high ambient CO_2_ level affects acid–base balance and bone metabolism in submarine crewmembers. Indeed, transitory (Schaefer 1979, 1982) or long-lasting (Pingree 1977; Messier et al. 1979) respiratory acidosis was evidenced in submariner populations during submersion as assessed by measurements of serum pH, Pco_2_, and bicarbonate concentrations. We recently completed these relevant data by demonstrating that submarine crewmembers experienced mild chronic respiratory acidosis (CRA) episodes (Holy et al. 2012) presenting some similarities with physiological deregulations encountered in patients suffering from chronic obstructive pulmonary disease (COPD).

Acidosis-driven alterations of bone metabolism in submariners were strongly suspected early in the 80s. Substantial modifications of calcium/phosphorus homeostasis were observed in crewmembers’ serum and urine over submersion (Gray et al. 1973; Davies and Morris 1979; Schaefer 1979, 1982; Dlugos et al. 1995; Luria et al. 2010; Holy et al. 2012). The development of serum and urine bone metabolism markers substantiated unequivocal evidence of bone metabolism alterations in submariners. As assessed by these serum markers, bone formation was markedly decreased in submarine crewmembers. Bone also contains an important stock of bases. Thus, bone demineralization leading to the release in the blood of these bases combined with calcium/phosphorus was construed as a physiological response to counterbalance hypercapnia-induced acidosis (Schaefer 1979, 1982).

During prolonged submarine patrols, the deprivation of sun exposure is another established cause for bone metabolism deregulation. It is widely acknowledged that 90% of the vitamin D body stock originates from UV-directed photosynthesis in the skin. In submariner populations, serum vitamin D status markedly decreased during prolonged patrols (Preece et al. 1975; Gilman et al. 1982; Dlugos et al. 1995; Luria et al. 2010; Holy et al. 2012), thus contributing to bone metabolism changes (Luria et al. 2010; Holy et al. 2012). Since the lack of sunlight exposure in winter can lead to vitamin D deficiency, we endeavored to ascertain the influence of seasonal vitamin D status on submariners’ bone metabolism and acid–base balance (Holy et al. 2012). We evidenced that the detrimental winter vitamin D status superimposed the hypercapnia negative influence on submariners’ bone metabolism (Holy et al. 2012).

Apart from acid–base balance and bone metabolism studies, rare publications have addressed to date the issue of submarine confinement effect over other organ metabolism such as muscles, liver, or kidneys. To the best of our knowledge, only two investigations have focused so far on renal function as assessed by measuring several parameters including urine excreted ions and metabolites (Gray et al. 1973; Dlugos et al. 1995). Urinary excretion of calcium, magnesium, phosphorus, sodium, sulfate, and uric acid fell during long-term submersion suggesting that submarine environment produces renal physiological changes (Gray et al. 1973; Dlugos et al. 1995).

The present study provides a screening of overall physiological regulations in submariners under prolonged patrols and seasonal changes. Urine and blood samples were collected from crewmembers assigned to a winter or summer patrol over 58 days. Various urine or serum metabolic markers including deoxypyridinoline (DPD), lactate, albumin, creatinine, nonesterified fatty acids (NEFA), and ionized sodium or potassium were quantified. Results yielded a valuable appraisal on main metabolic pathways modified by long-term submarine confinement correlated with seasonal changes.

## Materials and Methods

### Subjects and sample collections

Our investigations, herein, were carried out on account of the biological samples (urine and serum) collected in the submariner populations already studied by Holy et al. (2012). Briefly, 40 healthy submariners stood as volunteers for this study. The latter boarded on French ballistic missile submarines either at the end of summer (summer patrol or SP; *n *=* *20 submariners) or in mid-winter (winter patrol or WP; *n *=* *20 submariners). Data on the mean age and body weights of SP and WP crewmembers as well as records of mean CO_2_ levels and atmospheric pressure in both submarines are indicated in Table1. All subjects never received vitamin D supplementation during the last 4 months before boarding and during submersion. In addition, during submarine confinement, subjects answered a weekly questionnaire on their food habits and sport training frequency, conditions prone to modify bone metabolism. Indeed, high-phosphorus level (soda overconsumption) and calcium deprivation (lack of cheese and milk consumption) are known to be detrimental to bone metabolism, while sustained sport exercise is responsible for bone mass increase. None of the submariners exhibited markedly unbalanced food consumption or excessive physical activity.

**Table 1:** Patrol data related to submarines and crewmembers

Measured parameters	Summer patrol (SP)	Winter patrol (WP)
Submarine CO_2_ level (%)	0.72 ± 0.04	0.69 ± 0.06
Submarine atmospheric pressure (mbar)	999 ± 15	1026 ± 11
Crewmember age/prepatrol (years)	29 ± 1	31 ± 2
Crewmember weight/prepatrol (kg)	72.8 ± 2.2	73.6 ± 2.1
Crewmember weight/patrol day 58 (kg)	73.6 ± 2.2	73.8 ± 2.0

All values are expressed as mean ± standard error of the mean (SEM). Submarine CO_2_ and atmospheric pressure levels are mean of daily recorded values. The average age and weight were calculated with *n *=* *20 submariners for each patrol. Statistical analyses excluded significant difference between summer and winter patrols. Furthermore, submersion did not significantly modify crewmember weights (prepatrol vs. patrol day 58).

Urine and fasting venous blood samples were collected from submariners (within the first hour following awakening) before embarkation (prepatrol control values) and during submersion at patrol days 20, 41, and 58, respectively. Sera were prepared from blood samples in the shore and submarine sick bays and were stored at −20°C with urine samples for further metabolic marker analysis. However, measurements of ionized sodium (Na^+^) and potassium (K^+^) were carried out prior to serum aliquot freezing.

The protocol for this study has been registered at the French Ministry of Health and received official approval from the Ethical Committee known as “le Comité Consultatif de Protection des Personnes dans la Recherche Biomédicale (CCPPRB)” in Brest (France), in keeping with the ethical standards laid down in the 1964 Declaration of Helsinki.

### Measurements of urine and serum metabolic markers

Determinations of serum Na^+^ and K^+^ concentrations (in mmol/L) were performed on a Stat Profile pHOx Plus analyzer (Nova Biomedical, Les Ulis, France). The same apparatus was used for measurements carried out in the shore (prepatrol control value) or submarine sick bays (patrol days 20, 41, and 58). The reference ranges of values for healthy men are 135–145 and 3.5–5.0 mmol/L for serum Na^+^ and K^+^, respectively.

Urine inorganic phosphorus (Pi) and calcium (Ca) as well as serum lactate, NEFA (in mmol/L), total proteins, albumin (in g/L), and creatinine (in *μ*mol/L) were determined using a multiparametric ELAN autoanalyzer (Eppendorf, Germany) with appropriate kits (Diagnostica MERCK, Darmstadt, Germany). In addition, glomerular filtration rate (GFR) was estimated using the Modification of Diet in Renal Disease (MDRD) creatinine clearance calculation with an adjustment of body surface area at 1.73 m^2^ as previously described (Cirillo 2010). The MDRD creatinine clearance was expressed in mL/min. Serum reference values for healthy men at rest are 0.55–2.2 mmol/L for lactate, 0.126–0.445 mmol/L for NEFA, 32–50 g/L for albumin, 65–120 *μ*mol/L for creatinine, and 80–120 mL/min for MDRD creatinine clearance.

To evaluate bone resorption in urine, DPD level was measured with a pNPP-revealed competitive ELISA-immunoassay using Pyrilinks D kit (Pyrilinks D; Metra Biosystems, Mountain View, CA). The within- and between-run CVs were 5.6% and 6.3%, respectively. The amount of DPD was expressed in nmol/L.

### Statistical analyses

All values for serum or urine metabolic markers are expressed as mean ± standard error of the mean (SEM). Data were analyzed using a two-factor analysis of variance (ANOVA) with one between-group factor (winter vs. summer seasons) and one repeated-measure factor (submersion time). The interactions between both factors (season × submersion time) were also assessed. Whenever ANOVA was significant, post hoc comparisons were performed by LSD Fisher tests. For all statistical comparisons, data were analyzed with Statistica 7.1 software (StatSoft, Paris, France) and significance was set at *P *<* *0.05.

## Results

### Urine bone metabolic markers in submariners

**Figure 1:**
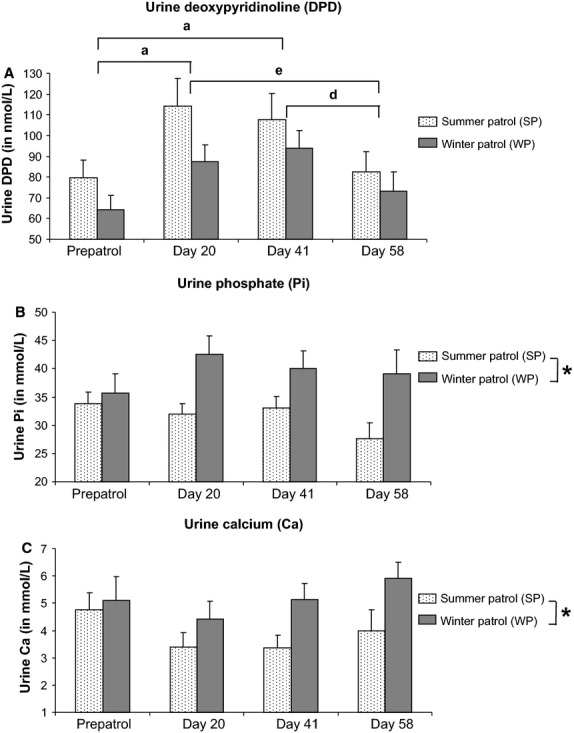
Urine bone metabolism markers in submariners. Deoxypyridinoline (DPD), inorganic phosphorus (Pi), and calcium (Ca) were considered as urine markers for bone resorption and bone metabolism. (A) DPD (in nmol/L), (B) Pi (in mmol/L), and (C) Ca (in mmol/L) were measured before (prepatrol) or during submersion (days 20, 41, and 58) in submariners assigned to summer (SP; *n *=* *20) or winter (WP; *n *=* *20) patrols. Data are expressed as mean ± SEM. Significant difference (*P *<* *0.05) when comparing ^a^submersion time to prepatrol time, ^d^day 58 with day 41; and ^e^day 58 with day 20. *Overall significant seasonal difference (*P *<* *0.05) between WP and SP groups.

In order to complete our previously published data (Holy et al. 2012), the present investigation focused on the three urine markers (DPD, Ca, and Pi) both strongly involved in bone metabolism (Fig.1).

Analysis of variance revealed a significant influence of submersion time (*P *<* *0.001) on urine DPD level without any influence of season (*P *=* *0.148) and without any interaction between season and submersion time (season × submersion time interaction; *P *=* *0.604). As shown in Figure1A, submersion induced a persistent increase in urine DPD measured in both SP and WP populations (between 35% and 45% increase for each patrol; *P *<* *0.01), up to patrol day 41. At the end of the patrol (day 58), a complete recovery of prepatrol urine DPD values was evidenced for both submarine crews.

Concerning urine Pi (Fig.1B), ANOVA indicated a seasonal influence (*P *<* *0.01) on this parameter without any effect of submersion time (*P *=* *0.449) and season × submersion time interaction (*P *=* *0.239). Regardless of the experimental time (prepatrol; patrol days 20, 41, and 58), urine Pi levels were consistently higher in WP submariners compared with their SP counterparts. The gap between WP and SP urine Pi concentrations ranged from +5% (prepatrol time) to +42% (patrol day 58). A similar seasonal effect (*P *<* *0.05) was observed with urine Ca concentrations for which WP urine Ca values were higher than SP urine Ca levels (Fig.1C), regardless of the submersion time. The difference between WP and SP ranged from +8% for prepatrol time to +48% for patrol day 58. ANOVA excluded a submersion time impact (*P *=* *0.163) and a season × submersion time interaction (*P *=* *0.626) on urine Ca concentrations.

### Serum ionic homeostasis in submariners

**Figure 2:**
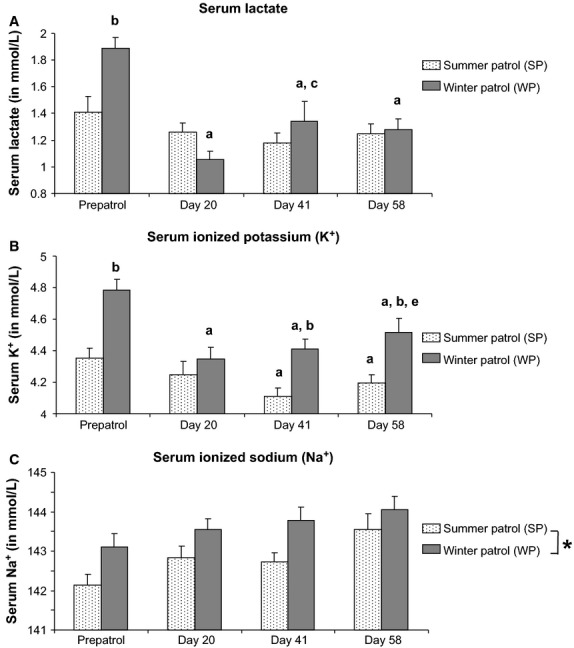
Serum ionic homeostasis in submariners. Serum concentrations (in mmol/L) of (A) lactate, (B) ionic potassium (K^+^), and (C) ionic sodium (Na^+^) were evaluated in submariners assigned to summer (SP; *n *=* *20) or winter (WP; *n *=* *20) patrols. Measurements were performed before (prepatrol) and during the submersion on days 20, 41, and 58, respectively. Data are expressed as mean ± SEM. Significant difference (*P *<* *0.05) when comparing ^a^submersion group with its respective prepatrol group; ^b^WP group to its respective SP group for a defined experimental time; ^c^day 41 with day 20 within a same patrol; and ^e^day 58 with day 20 within a same patrol. *Overall significant seasonal difference (*P *<* *0.05) between WP and SP groups.

Analysis of variance revealed a significant season × submersion time interaction for lactate (*P *<* *0.01). The only significant difference in lactate levels between WP and SP was observed for prepatrol values for which WP lactate was 34% higher than SP (*P *<* *0.01). Regarding SP, serum lactate concentrations remained unaltered throughout the submersion period. For WP, a 56% decrease was evidenced over the first 20 days of submersion (*P *<* *0.001) and this value remained even up to the end of the patrol (Fig.2A).

For serum K^+^ levels, ANOVA demonstrated a significant season × submersion time interaction (*P *<* *0.05). As shown in Figure2B, WP serum K^+^ concentrations consistently exceeded SP K^+^ values (between 7.2% and 9.9% higher) in prepatrol conditions and at submersion days 41 and 58. For SP, a 5.6% reduction in K^+^ level was detected at patrol day 41 (*P *<* *0.01 compared to prepatrol value). This low K^+^ concentration remained consistent over the rest of the submersion period. In WP, a 9.1% decrease in serum K^+^ was measured as early as patrol day 20 (*P *<* *0.01) compared to prepatrol level (Fig.2B). Such a decrease remained even up to the end of the patrol despite a weak increase in K^+^ concentration on patrol day 58 (3.9% increase compared to patrol day 20 value; *P *<* *0.05).

Analysis of variance exhibited a significant effect of both season (*P *<* *0.05) and submersion time (*P *<* *0.001) on serum Na^+^ without any significant season × submersion time interaction (*P *=* *0.737). As shown in Figure2C, throughout the submersion period Na^+^ level increased slightly compared to control value recorded in either WP (0.3% increase; *P *<* *0.05) or SP groups (1.0% increase; *P *<* *0.001). The serum Na^+^ concentration for WP was higher than for SP regardless of the experimental time under specific scrutiny.

### Serum protein metabolic markers in submariners

For total proteins and NEFA, ANOVA revealed neither any seasonal effect (*P *=* *0.466 for total proteins and *P *=* *0.807 for NEFA) nor of submersion time (*P *=* *0.283 for total proteins and *P *=* *0.163 for NEFA) on both parameters underscored without any significant season × submersion time interaction (*P *=* *0.193 for total proteins and *P *=* *0.803 for NEFA) (data not shown).

**Figure 3:**
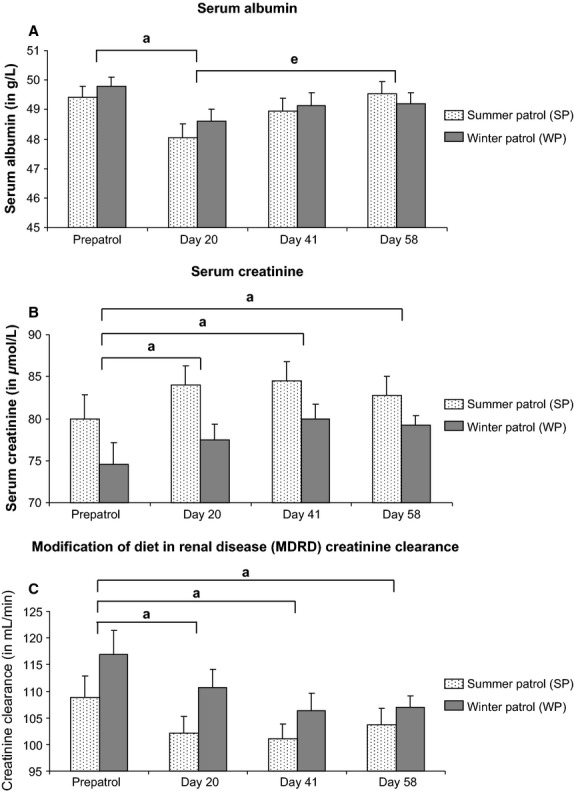
Serum proteins in submariners. Venous blood samples were collected from submariners assigned to summer (SP; *n *=* *20) or winter (WP; *n *=* *20) patrols. Serum albumin (A) and creatinine (B) were measured before (prepatrol) and during the submersion (days 21, 40, and 58). The Modification of Diet in Renal Disease (MDRD) creatinine clearance (C) was calculated with an adjustment of body surface area at 1.73 m^2^ in order to evaluate the glomerular filtration rate. Serum albumin, serum creatinine, and MDRD creatinine clearance were expressed in g/L, *μ*mol/L, and mL/min, respectively. Data are expressed as mean ± SEM. Significant difference (*P *<* *0.05) when comparing ^a^submersion time to prepatrol time and ^e^day 58 with day 20.

Even if total proteins remained unmodified, two specific protein levels (i.e., albumin and creatinine) were affected by the submersion. For albumin levels, ANOVA exhibited a significant effect of submersion time (*P *<* *0.001) on this serum parameter without any influence of season (*P *=* *0.614) and season × submersion time interaction (*P *=* *0.551). As shown in Figure3A, a transient significant 3% decrease in albumin was measured at patrol day 20 for both patrols compared to prepatrol levels (*P *<* *0.05). For serum creatinine concentration (Fig.3B), statistical analysis revealed a significant effect of submersion time (*P *<* *0.001) on this parameter without any influence of season (*P *=* *0.086) and season × submersion time interaction (*P *=* *0.509). Creatinine level rose by about 5% at the beginning of WP and SP patrols (day 20) and this increase was sustained up to the end of the submersion period (*P *<* *0.05). In terms of GFR, ANOVA indicated an influence of submersion time (*P *<* *0.001) on MDRD creatinine clearance without any impact of season (*P *=* *0.323) and season × submersion time interaction (*P *=* *0.154). Submersion induced a steady lowering in MDRD creatinine clearance throughout the entire patrol for both WP (about 8% decrease) and SP (about 6% decrease) submariners (Fig.3C).

## Discussion

Urine and serum samples analyzed in the present study were collected in SP and WP submariners who joined an extensive published investigation addressing the influence of seasonal vitamin D deficiency and submarine confinement-induced hypercapnia on bone metabolism (Holy et al. 2012).

### Urine bone metabolism markers are influenced by both season and submersion

We elected to probe, herein, urine Pi, Ca, and DPD with a view to enhancing further our data on submariners’ bone metabolism as previously assessed by serum marker analyses (Holy et al. 2012).

Deoxypyridinoline is widely acknowledged as a urine marker used to quantify bone resorption (Robins et al. 1994). Regardless of the boarding season, submersion induced a transient rise in urine DPD concentrations in submariners, up to patrol day 41. This finding implies that a substantial bone resorption process occurred in WP and SP crewmembers over the first 41 days of deployment. Urine DPD analysis confirmed the bone resorption time course pattern we already observed in the same WP and SP submariner populations using a serum bone resorption marker, namely, COOH-terminal telopeptide of type I collagen or ICTP (Holy et al. 2012). There is a significant linear correlation (*r *=* *0.67; *P *<* *0.0001) between urine DPD and serum ICTP level in submarine crewmembers, regardless of the considered experimental time and boarding season (*n *=* *160 by gathering together data values from all WP and SP submariners at all experimental times). To the best of our knowledge, only one other study focused on bone resorption marker analysis in submariners (Luria et al. 2010). Contrary to our current results, the latter observed a drop in bone resorption marker levels including tartrate-resistant acid phosphatase activity (TRAP5b) and type I carboxy-terminal telopeptide (CTx) in crewmembers’ serum after a 30-day patrol (Luria et al. 2010). The reason for such a data discrepancy may likely result from the type of the urine bone resorption markers used by Luria’s team and us. Indeed, relative abundance of ICTP and CTx in urine or serum varies depending on the nature of the considered bone metabolism alteration. Using both in vitro and in vivo approaches, Garnero et al. (2003) evidenced that cathepsin K-induced bone collagenolysis released large amounts of CTx but did not allow a detectable release of ICTP. On the contrary, bone collagen degradation triggered by matrix metalloproteinases (MMPs) fostered ICTP release, however, without any CTx liberation. Therefore, we may legitimately contend that bone resorption induced by submarine confinement is likely to result from a MMP-collagenolytic pathway leading to ICTP liberation (Holy et al. 2012) without CTx release (Luria et al. 2010).

Prior to the development of specific bone metabolism markers, bone resorption in humans was indirectly estimated by the levels of either calcium or Pi excreted in urine or alternatively by the concentrations of these ions in serum. Indeed, bone represents roughly 85% of phosphorus and 98% of calcium body stocks. Hence, bone resorption obviously leads to a substantial release of both components into serum and subsequently into urine (DuBose 2001). Concerning Pi and Ca levels in serum, an almost consensual increase in Pi and Ca concentrations was demonstrated in submariners subjected to patrols lasting up to 68 days (Gray et al. 1973; Messier et al. 1979; Dlugos et al. 1995; Holy et al. 2012). Such a finding is congruent with the bone resorption rise revealed in our submarine crewmembers as discussed earlier. However, in most of these studies, submersion induced an unexpected reduction in both urine Pi and Ca (Gray et al. 1973; Messier et al. 1979; Dlugos et al. 1995), whereas hyperphosphaturia and hypercalciuria should normally be favored on account of the observed Pi and Ca accumulation in serum. According to the statistical analysis of our present data, submarine confinement did not modify urine Pi and Ca levels. However, we evidenced seasonal variations on both urine Pi and Ca concentrations. Moreover, submersion seemed to exacerbate these urine Pi and Ca seasonal changes. Both hyperphosphaturia and hypercalciuria were preferentially encountered in our winter-deployed submariners. Such an observation is consistent with the increased bone resorption measured in WP crewmembers compared with SP population using urine DPD (our present data) or serum ICTP (Holy et al. 2012) markers. Ours results are also consistent with an annual epidemiologic survey focusing on seasonal variations in urinary composition performed on 342 male volunteers living in the United Kingdom (Rose and Westbury 1979). In this study, urine Pi excretion was 12% higher in winter (March/April) than in the end-summer period (September/October). Nevertheless, seasonal variation in urine Ca remained more controversial since either winter (Marya et al. 1982) or summer (Robertson et al. 1977) hypercalciuria were indifferently observed. According to Robertson et al. (1977), seasonal variation in urine Pi and Ca levels may stem from the effect of sunlight-synthesized vitamin D on intestinal absorption of both phosphorus and calcium rather than the dietary intake of these ions.

Altogether our present urine-related bone metabolism data unequivocally evidenced detrimental bone resorption activity in both our WP and SP submariners. These results confirmed the bone metabolism alteration we previously demonstrated in the same submarine crewmember populations using serum bone metabolism markers (Holy et al. 2012). The question that can be addressed is the clinical relevance of the altered bone metabolism detected in submariners. Using quantitative ultrasound measurements of tibial speed of sound, submariners subjected to a 1-month submersion exhibited significant decrease in bone strength at 4 weeks after return to shore, but bone strength returned to baseline levels at the 6-month follow-up (Luria et al. 2010). In an extensive epidemiologic survey, Gasier et al. (2014) measured by DXA the bone mineral content and density in both lumbar spine and dual proximal femur of 462 U.S. Navy submariners and confirmed that submergence up to 3 months do not appear to compromise skeletal health.

### Serum ionic homeostasis in submariners is only influenced by seasons

Even though a valuable seasonal influence has been clearly identified on acid–base balance parameters and bone metabolism markers in crewmembers during submersion (Holy et al. 2012; our present data), the boarding season however bore minimal impact over other serum metabolic markers scrutinized in our submariner populations. Among these metabolism markers, only serum lactate, Na^+^, and K^+^ levels were altered by the boarding season.

The seasonal differential impact over serum lactate concentrations was observed only under prepatrol conditions. Before boarding, WP submariners exhibited a slight lactic acidosis but the submersion rapidly normalized serum lactate levels. For SP crewmembers, serum lactate concentrations remained within the standard value range either in prepatrol conditions or during submarine confinement. Interestingly, a 1-year monitoring of various blood metabolic markers in young Russian adults living near the 62° Northern latitude evidenced a hyperlactatemia during winter months (between September and March) (Kochan and Eseva 2009). Authors argued that winter lactic acidosis could be induced by an anaerobic glycolysis resulting from a seasonal adaptation to maintain energy homeostasis whenever air temperature dropped to negative values and daylight span decreased (Kochan and Eseva 2009). Hence, the high serum lactate concentration exhibited by our WP submariners in prepatrol conditions most likely results from a common seasonal adaptation from the body energy metabolism. A further asset from our data rests with disclosed evidence of a swift normalization process with regard to serum lactate levels in WP submariners at the beginning of the submersion stage. Such a finding provides evidence that submarine microenvironment triggers rapid physiological changes prone to counterbalance WP crewmembers’ lactic acidosis. Several investigations (Pingree 1977; Messier et al. 1979; Holy et al. 2012) clearly demonstrated that submarine-driven hypercapnia induced CRA in submariners. One of the major consequences of respiratory acidosis is the bicarbonate accumulation in blood, a feature which has been already assessed in submariners (Holy et al. 2012). Bicarbonate is an efficient physiological base and its accumulation in blood could prevent lactic acidosis thus accounting for the swift normalization effect of serum lactate levels in our WP crewmembers.

Concerning K^+^ and Na^+^, we clearly identified an influence of the boardng season on the serum concentrations of these two cations, but the trend of their concentrations during submersion varied according to the considered ion.

With regard to prepatrol conditions, both serum Na^+^ and K^+^ levels were significantly higher in WP crewmembers compared to their SP counterparts. Identical seasonal changes in serum cation concentrations were pointed out in Japanese healthy male students (Morimoto et al. 1969). These authors demonstrated that both serum K^+^ and Na^+^ levels increased in winter as compared to summer values. A similar seasonal influence over serum lactate concentrations has been underscored here-above in our current article. Furthermore, a significant linear correlation (*r *=* *0.550; *P *<* *0.001) was shown between prepatrol serum lactate and serum K^+^ levels in submariners, regardless of the boarding season (*n *=* *40 by gathering together data values from all SP and WP submariners at prepatrol time). The close-knit correlation between elevated serum lactate and K^+^ concentrations in our WP population suspected to suffer from a slight seasonal metabolic acidosis remains noteworthy. Usually, in pathological metabolic acidosis associated with lactic acidosis, serum potassium level was not increased (Kraut and Madias 2010). On the contrary, metabolic acidosis related to increases in serum K^+^ concentrations are classified as hyperkalemic hyperchloremic acidosis and serum lactate generally remained in the standard value range (Kraut and Madias 2010). Thus, the slight seasonal lactatemia correlated with hyperkaliemia encountered in our WP submariners before boarding did not match pathological metabolic acidosis patterns classically described in the literature. The reasons for such a discrepancy need to be investigated further.

During submersion, a continuous rise in serum Na^+^ concentrations was evidenced in both SP and WP populations. As a matter of interest, the evolution of serum sodium levels in submariners during prolonged patrols is comparable to the changes in serum bicarbonate levels previously detected in the same crewmember populations (Holy et al. 2012) despite the absence of statistical correlation between both biological markers.

In WP submariners, prolonged patrols induced a rapid decrease in serum K^+^ level as early as patrol day 20, paralleling the evolution of lactate when WP crewmembers were subjected to submarine confinement. For longer winter patrol times, a slight increase in potassium level was measured in WP submariners’ serum. For SP crewmembers, a drop in serum K^+^ concentration was also detected but it occurred later during the submersion period (patrol day 41). Other investigators obtained similar serum K^+^ and Na^+^ data in submariner populations submitted to prolonged patrols extending to 60 days (Messier et al. 1979; Schaefer 1979). Indeed, submarine confinement induced plasma sodium increase concomitantly with plasma potassium decrease (Messier et al. 1979), a finding which is consistent with the results yielded within our study. Furthermore, these authors also demonstrated that such plasma cation variations paired up all at once with a distinct red blood cell K^+^ drop as well as a significant increase in red blood cell Na^+^ suggesting thereby hypercapnia-driven changes in red blood cell permeability (Messier et al. 1979). According to Messier et al. (1979), these modifications in red blood cell permeability are generally considered as evidence of active pump transport changes.

### Serum protein metabolic markers are only affected by submersion

In terms of serum protein levels, total protein concentrations were neither modified by the season nor by the submersion time span in our crewmember populations. Nevertheless, we focused our investigations on serum albumin and creatinine, two proteins considered as main markers for liver and kidney metabolisms, respectively.

For serum albumin, only a slight 3% transient decrease in this protein concentration was observed in submariners after 20 days of submersion and regardless of the boarding season. Since in vivo serum albumin half-life amounts to 19 days, we may hence legitimately infer that the alteration of albumin level evidenced on patrol day 20 result from a hepatic metabolism modification induced by the submarine confinement and that this modification occurred very early in the patrol time course. To date, a single study has reported changes in serum albumin quantities in submariners after a prolonged patrol (Luria et al. 2010). A slight 6% increase in serum albumin was shown in submariners following a 30-day patrol (Luria et al. 2010), a finding that differed from our data. It is commonly acknowledged that a rise in serum albumin content stems from a hemoconcentration related to dehydration (DuBose 2001) which could be the physiological event occurring in Luria’s crewmember population.

Concerning the well-characterized kidney metabolism marker, namely serum creatinine, we demonstrated a slight increase in this protein in submariners’ sera within the first 20 days of submersion. Moreover, the elevated creatinine level remained steady up to the end of the patrols, regardless of the boarding season. This serum creatinine rise was associated with a decrease in MDRD creatinine clearance thus providing further grounds with regard to an observable modification in renal metabolism induced by submersion-related hypercapnia. To date, only two publications focused on submariners’ kidney functions (Gray et al. 1973; Dlugos et al. 1995). Based on the measurements of serum and urine Ca^2+^, Mg^2+^, Pi, and H^+^ levels, Gray et al. (1973) suggested that exposure to CO_2_ in submarine altered the renal handling of H^+^ and Ca^2+^ ions. In an extensive study, Dlugos et al. (1995) evidenced significant decreases in daily urinary excretion of calcium, creatinine, uric acid, sodium, sulfate, and phosphorus in submariners subjected to a 68-day patrol. Despite kidney metabolism changes, Dlugos concluded that exposure to submarine environment induces physiologic modifications (i.e., decrease in vitamin D level) that prevent renal stone formation (Dlugos et al. 1995). However, a health data analysis carried out over 10 years of Polaris submarine patrols (Tansey et al. 1979) demonstrated that submariners tended to display a higher genitourinary illness rate compared to surface U.S. Navy population. Investigators suggested that increased CO_2_ levels in the submarine atmosphere may have contributed to the higher incidence of ureteral calculi in submarine personnel (Tansey et al. 1979). Furthermore, hypercapnia animal models substantiated the potential nephrolithiasis risk induced by CO_2_ challenging (Schaefer et al. 1979a,b). Indeed, the exposure of guinea pigs to elevated CO_2_ levels ranging between 0.5% and 1.5% up to 8 weeks induced kidney calcification as assessed by kidney calcium dosing and microscopic histology (Schaefer et al. 1979a,b).

We previously paralleled CRA episode and bone metabolism alterations exhibited in our submariner populations with similar physiologic deregulations presented by COPD patients (Holy et al. 2012). Such a parallel between submariners and COPD patients may be again legitimately hypothesized in terms of kidney metabolism. Indeed, chronic renal failure is the second most common comorbidity (26.3%) after hypertension (64.2%) in COPD patients (Incalzi et al. 2010; Terzano et al. 2010). In addition, an increased concentration in serum creatinine was demonstrated in COPD patients affected by CRA (Gonlugur and Gonlugur 2007). Such an observation is consistent with the weak accumulation of serum creatinine we evidenced in our submariner population exhibiting CRA episode triggered by high CO_2_ level exposure during prolonged patrols (our present results and Holy et al. 2012).

Taking into account all the kidney-related data here-above, we are entitled therefore to address the following issue, namely whether or not a potential risk for nephrolithiasis stands as a potential health hazard for submarine crewmembers accumulating several ballistic missile submarine assignments over a few years’ time.

## Conclusions

Our investigation herein confirmed the bone metabolism alteration previously shown in submarine crewmembers subjected to prolonged patrols (Luria et al. 2010; Holy et al. 2012). Using a urine bone resorption marker (DPD), we demonstrated a bone resorption increase triggered by submersion-induced hypercapnia. Moreover, as a breakthrough contribution we assessed the influence of both submarine confinement and boarding season on various metabolic markers in submariners’ serum. On the basis of the appraisal of serum albumin concentrations, we suspected a tiny transitory modification in liver metabolism occurring within the first 20 days of submersion. More importantly, measurements of serum creatinine level and calculation of MDRD creatinine clearance evidenced weak changes in submariners’ renal physiology. This metabolic disturbance is likely to foster kidney stone formation in crewmembers accumulating submarine assignments over a short period of time. Quantifications of serum Na^+^ and K^+^ concentrations revealed possible changes in active ionic pump transport in submariners and these changes varied depending on the boarding season. As a final point, submersion provided beneficial conditions prone to counterbalance seasonal lactic acidosis detected in crewmembers boarding in winter season.
